# Effects of dexmedetomidine on oxidative stress, programmed cell death, liver function, and expression of peripheral immune cells in patients with primary liver cancer undergoing hepatectomy

**DOI:** 10.3389/fphys.2023.1159746

**Published:** 2023-04-11

**Authors:** WenYing Li, MingHao Chen, YuXin Gong, Feng Lin, Chen Sun

**Affiliations:** Department of General Surgery, The Second Affiliated Hospital of Harbin Medical University, Harbin, Heilongjiang, China

**Keywords:** dexmedetomidine, oxidative stress, programmed cell death, primary liver cancer, immune

## Abstract

**Study background:** Primary liver cancer is a severe health issue that imposes a significant health burden on families. Oxidation and subsequent cell death impair liver function and provoke an immune response. The present article investigates the effect of Dexmedetomidine on oxidation, cell death, the expression of peripheral immune cells, and liver function. The clinical data will represent the facts and evidence of the effects of this intervention.

**Methods:** We analyzed clinical data reporting various accounts of the effects of Dexmedetomidine on oxidation, cell death, the expression of peripheral immune cells, and liver function among patients who underwent hepatectomy. The surgical procedure reported the differences in cell death as procedural outcomes among pre- and post-treatment records were compared and contrasted.

**Results:** We found decreased cell apoptosis in the treatment group: the number of incisions to remove dead cells was lower in the treatment group than in the pre-treatment group. Likewise, lower oxidation was reported in pre-treatment than in post-treatment records. The expression of peripheral immune cells was higher in the pre-treatment clinical data than in post-treatment, suggesting a reduction in oxidation following dexmedetomidine treatment. Liver function was a function of oxidation and cell death outcomes. In the pre-treatment clinical data, liver function was poor, whereas improved functions were reported in the post-treatment clinical data.

**Discussion:** We found compelling evidence of Dexmedetomidine’s effects on oxidative stress and programmed cell death. The intervention suppresses the production of reactive oxygen species and the consequential apoptosis. Additionally, liver functions improve due to the decrease in hepatocyte apoptosis. Since the peripheral immune cells are expressed against tumors, a decrease in the progression of primary liver cancer decreased the expression of the peripheral immune cells.

**Conclusion:** Dexmedetomidine’s positive effects stood out in the present research article. The intervention reduced oxidation by balancing the production of reactive oxygen species and the detoxification processes. Reduced oxidation induced reduced cell death through apoptosis, resulting in a low expression of peripheral immune cells and improved liver functions.

## Introduction

Primary liver cancer is the sixth most diagnosed cancer and fourth in mortality rate worldwide (Dasgupta et al., 2020). In 2018, approximately 841,000 people were diagnosed with primary liver cancer (a rate of 9.3 cases per 100,000 person-years), while 782,000 deaths were reported, estimated to be a mortality rate of 8.5 per 100,000 person-years. By 2040, the total number of people living with cancer will be 1.4 million, out of which the incidence of primary liver cancer is expected to rise by 55% (Rumgay et al., 2022a) and will account for 80% of all cancer cases.

The high incidence of the disease and its incurable nature pose a significant health concern worldwide. Primary liver cancer is rapidly becoming a common topic in various sectors of the medical fraternity and among patients. Different medications have been designed; further studies are ongoing to find a curative agent. The effects of medications indicated for primary liver cancer or any other disease are key in clinical practice. Adverse or positive effects are essential when indicating a drug for a particular disease. A drug is indicated for a particular disease if it produces minimal adverse effects and maximum therapeutic effects.

The literature shows that Dexmedetomidine has been extensively used among primary liver patients undergoing hepatectomy (Chen et al., 2020). The mechanisms of drugs within biological systems can either trigger the progression or suppression of the disease. In discussing the effects of Dexmedetomidine in primary liver cancer, an investigation of the influence of the drug on biological processes and macromolecules is necessary. Additionally, effects on cellular and organ functions are essential. Concerning the present study, resulting oxidative stress, programmed cell death, liver functions, and expression of immune cells become essential elements of the discussion.

The effects of Dexmedetomidine in primary liver cancer remain unclear. The literature on primary liver cancer management reports mixed outcomes: Dexmedetomidine promotes the progression of primary liver cancer and suppresses its development (Liu P et al., 2020; Zhou et al., 2022). The present article investigates the effects of Dexmedetomidine on the expression of peripheral immune cells, oxidative stress, and programmed cell death, alongside liver functions among primary liver cancer patients who underwent hepatectomy. The pathology of primary liver cancer entails the immune system, *via* the peripheral immune cells and liver functions, as fundamental outcomes, alongside oxidation and programmed cell death. The effect of Dexmedetomidine will inform the status of the drug’s efficacy and safety and the resulting clinical practices in managing primary liver cancer.

Primary liver cancer unfolds from a multi-step biological process, including oxidation processes and cell death through various mechanisms, alongside the expression of immune cells and liver functions (Ho et al., 2016; Gastrointestinal Cancer, 2022). The primary objective of this article is to report the outcomes of Dexmedetomidine intervention on these processes. The outcomes of the present study will be crucial for clinical practice and inform studies and research on primary liver cancer treatment. Oxidative stress and programmed cell death are two fundamental aspects of primary liver cancer. Hepatectomy is performed to remove dead and affected cells (Orcutt and Anaya, 2018). Oxidative stress causes intracellular and extracellular dysbiosis, resulting in the disruption of homeostasis. The latter is a crucial event in affecting liver functions. Cell death is imminent when the reactive oxygen species are produced and exceed antioxidant functions and processes (Lobo et al., 2010). Dexmedetomidine suppresses oxidative stress, reduces programmed cell death, and influences the expression of peripheral immune cells and liver functions among patients with primary liver cancer.

## Materials and methods

### Data collection

Oxidative stress and programmed cell death are key processes in patients with primary liver cancer. To understand the effects of Dexmedetomidine on oxidative stress and programmed cell death among patients with primary liver cancer, it is important to define oxidative stress and its role in primary liver cancer, as well as to define programmed cell death and its associations with oxidative stress in primary liver cancer.

Accurate definitions of oxidative stress and programmed cell death will be obtained from authentic sources such as databases, including the American Cancer Society and the National Cancer Institute, which provide contextualized and accurate definitions of primary liver cancer. Peripheral immune cells in the disease mechanism and the role of oxidative stress and programmed cell death will also be explored.

We will review literature on the association between oxidative stress and programmed cell death and contrast the findings with the data collected in the present study. The surgical procedure to remove malignant or benign sections provided fundamental data on cell death and oxidation. We will review patient follow-up on patients diagnosed with primary liver cancer who underwent the surgical procedure. Post-surgical outcomes such as recovery and the health status of the patients will be reviewed to determine the effects of Dexmedetomidine on oxidative stress and programmed cell death. The health status of the cells will be reviewed regarding Dexmedetomidine’s effects on oxidative stress and programmed cell death. The appendix summarizes the number of patients who underwent hepatectomy before and after treatment to remove dead cells. Dexmedetomidine intervention decreased cell death by suppressing oxidation.

To differentiate the effects of Dexmedetomidine among patients who underwent hepatectomy, we will contrast the effects of Dexmedetomidine in the group that underwent the surgical procedure against the group that did not undergo the procedure. We aim to observe the differences in the outcomes of the intervention. We will collect evidence and information on the nature of the cells and oxidative stress. Information on the differences in oxidative stress and programmed cell death will be analyzed to determine the effect of the intervention. (All data collection is conducted in some hospitals in China, Based on our commitment to relevant institutions, we have not published their specific addresses and names).

### Liver function

Liver function is a fundamental aspect to consider when assessing patients with primary liver cancer. Medications that are metabolized by the liver are not recommended for individuals with liver complications as it can lead to the accumulation of metabolites. In this study, we will examine the impact of Dexmedetomidine on liver function among patients with primary liver cancer.

To assess the effect of Dexmedetomidine on liver function, we will compare patients who received the treatment to those who did not. We will review clinical information to establish whether the medication has a positive impact on liver function. Blood test results, including levels of vital biomolecules such as albumin, total protein, alkaline phosphatase, bilirubin, lactate dehydrogenase, and prothrombin time (Supplementary Material), will be analyzed and compared to ideal levels within homeostatic levels. This comparison will inform us whether liver functions have improved.

The reduction in the number of patients experiencing oxidation and cell apoptosis indicate the positive effects of Dexmedetomidine on hepatectomy. Any changes in the ideal levels of biomolecules suggest a disruption of biological systems, often resulting from cell death. Programmed cell death and apoptosis caused by oxidative stress can alter the levels of vital biomolecules (see Supplementary Material).

Furthermore, we will establish a connection between programmed cell death, oxidative stress, and Dexmedetomidine to determine the impact of the medication on liver function among primary liver cancer patients undergoing hepatectomy. Our study has revealed that Dexmedetomidine intervention suppresses oxidative stress and, subsequently, programmed cell death. We will compare liver function reports before and after the intervention to assess the impact of Dexmedetomidine on liver function.

## Results

### The effects of dexmedetomidine on oxidative stress and programmed cell death

The American Cancer Society defines primary liver cancer as “cancer that starts in the liver” (American Cancer Society, 2019; National Cancer Institute, 2019). In simple terms, the disease forms in the liver cells: The malignant or cancer cells establish in hepatocytes. This means that the cancer cells do not spread to hepatocytes from other organs or tissues of the body. The literature search revealed that cholangiocarcinoma and hepatocellular carcinoma are the main types of primary liver cancer (Cancer Research UK, 2021). However, hepatocellular carcinoma is the most prevalent primary liver cancer among adults. Hepatocellular carcinoma, the most prevalent form of primary liver cancer, holds crucial information on oxidative stress and programmed cell death. At least 90% of hepatocellular carcinoma is characterized by inflammation and fibrosis (García-Pras et al., 2021), which leads to the generation of reactive oxygen species and oxidative stress (Fu and Chung, 2018). Inflammation and fibrosis indicate the expression of peripheral immune cells and liver functions, respectively. Like any other disease, primary liver cancer must trigger immune functions initiated by oxidative stress and programmed cell death.

We found different types of programmed cell death, necroptosis, apoptosis autophagy, and pyroptosis (García-Pras et al., 2021), featuring in the progression of hepatocellular cancer. Programmed cell death in primary liver cancer is associated with chronic inflammation through an intrinsic relationship (Table 1). Oxidative stress accelerates liver carcinogenesis and fibrogenesis, cell inflammation, and death. Inflammatory cell death is the main feature of primary liver cancer (García-Pras et al., 2021). The association of oxidative stress, programmed cell death, and the subsequent effect of Dexmedetomidine is founded on inflammation. A study by Uchida et al. (2020) reported that inflammation-induced the production of reactive oxygen species and hepatocyte apoptosis. The effects of Dexmedetomidine are evident in the liver cells through dead cells. Dead hepatocytes are evident among primary liver cancer patients undergoing hepatectomy, as apoptosis leads to the death of the affected cells.

**TABLE 1:** A summary of Dexmedetomidine’s effects on immune cells. Adapted from R. Chen et al., “Effects of Dexmedetomidine on Immune Cells: A Narrative Review,” Frontiers in Pharmacology, vol. 13, no. 3 May 2022, doi: 10.3389/fphar. 2022.829951.

Target cells	Effects	
	Suppression	Induction
Dendritic cells	Proinflammatory cytokines (TNF-alpha, IL-1beta, IL-6, IFN-gamma), Immunomodulatory factor (IL-12, IL-23), Class II MHC, and costimulatory molecules (I-Ab and CD86)	Anti-inflammatory cytokine IL-10
Natural Killer cells	Development and metastasis of tumors	Increase the number and maintain the activity
Eosinophils mast cells	Chemokines (eotaxin), Degranulation Proteolytic enzyme MMP-9	Proteolytic enzyme MMP-2
Neutrophils	Proinflammatory cytokines (TNF-alpha, IL-6, Necrosis Factor), Antimicrobial effectors (ROS, RNS, NO, iNOS), Respiratory eruption, Local aggregation of neutrophils	Elimination of pathogen
Monocytes	The ratio of CD42+/CD14+ Pro-inflammatory cytokines (IL-6, TNF-alpha), The expression of Cx43, PKC-alpha, VLA-4 and LFA-1), Monocyte endothelial cells adhesion	The ratio of HLADR+/CD14+
Macrophages	Pro-inflammatory cytokines (IL-6, COX-II, PGE2, HMGBI), Inflammatory protein MiP-2	TNF-alpha, IL-1beta, transforming growth factor TGF-beta1, Anti-inflammatory cytokine (IL-10), The production of Th1 cells by promoting the secretion of IL-12, Polarization of M2, Clearance of Neutrophil and autophagy of mitochondria
B Cells		Chemokine (IL-2)
T Cells	The amount of CD8^+^, CD3^+^, CD4^+^, CD4+/CD8+	Proinflammatory cytokines (IL-17A) Immune regulatory factors (IFN-gamma)

### The effects of dexmedetomidine on liver function

Liver functions are significantly influenced by oxidative stress and programmed cell death, meaning every aspect affecting oxidative stress and programmed cell death indirectly affects liver functions. We began by linking liver functions with the effect of Dexmedetomidine on oxidative stress and programmed cell death. The protection of liver cells, and the organ as a whole, was a prominent observation. Dexmedetomidine’s effects on oxidative stress and programmed cell death all pointed to protective effects on liver cells and organs, implying the conservation of liver function. Conservation and improvement of liver functions unfolded in different ways.

Improved liver functions could be pointed at the suppression of oxidative stress and reduction in programmed cell death. After reviewing pre- and post-treatment data, we found a significant difference in liver function in the treatment and control groups. Dexmedetomidine’s suppressive action on oxidative stress by inhibiting H_2_O_2_-induced apoptosis prompts vital revelations on liver functions. Logically, cell death undermines organ function. Therefore, the death of liver cells would undermine liver function. When the process causing cell death has been suppressed, liver function is expected to improve. This notion concurs with the present study’s findings, per evidence obtained from pre- and post-treatment information. DNA damage is central to cell functions and, subsequently, organ function.

The reaction between the reactive oxygen species and important. Pre-treatment evidence reported high DNA damage courtesy of the interaction between the reactive oxygen species and important macromolecules alongside DNA damage. Dexmedetomidine’s effect downregulates the production of reactive oxygen species, implying a balance between the reactive oxygen species and the antioxidant processes. Basic physiological principles indicate that organ function declines with DNA damage. In this context, loss of liver function would occur in two ways: The interaction between the reactive oxygen species and DNA damage. Cellular functions would be lost when the reactive oxygen species react with biomolecules like proteins and lipids. Eventually, basic functions like energy production and supply to liver cells would be lost. DNA damage leads to loss of coordination of cellular functions. However, dexmedetomidine treatment inhibits the oxidative stress processes, which restores homeostatic conditions. Restoration of homeostasis is key to the re-introduction of optimum liver functions.

### Analysis

#### Dexmedetomidine’s effects on oxidative stress and programmed cell death

**FIGURE 1:**
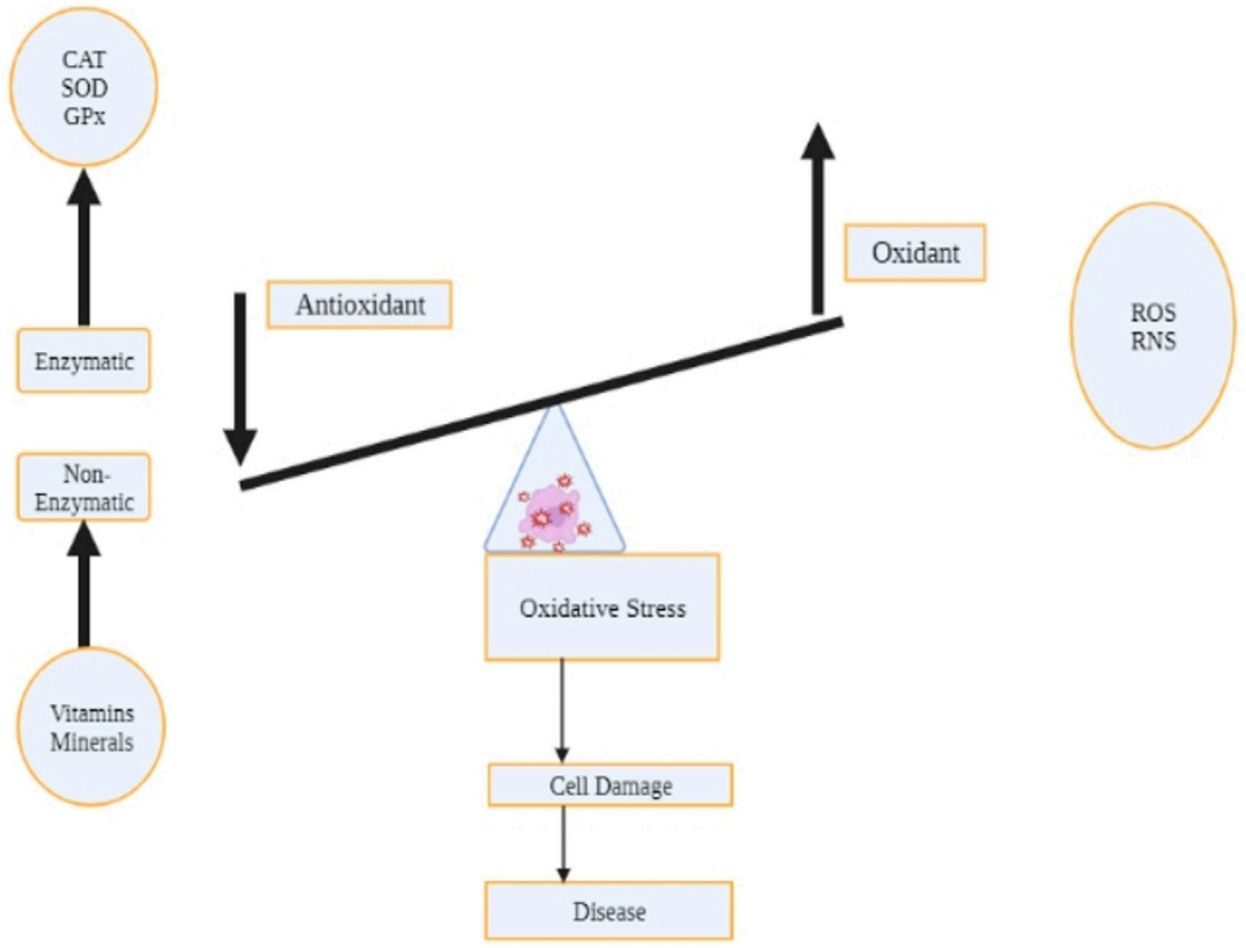
The status of oxidative stress hangs on the balance of the production of the reactive oxygen species and the antioxidant processes and biological compounds. Cell damage due to oxidation results from the downregulation of non-enzymatic compounds (minerals and vitamins) facilitating antioxidant functions, decreasing reactive oxygen species production. An increase in antioxidant processes and biological compounds decreases ROS production, and the converse is true.

**FIGURE 2:**
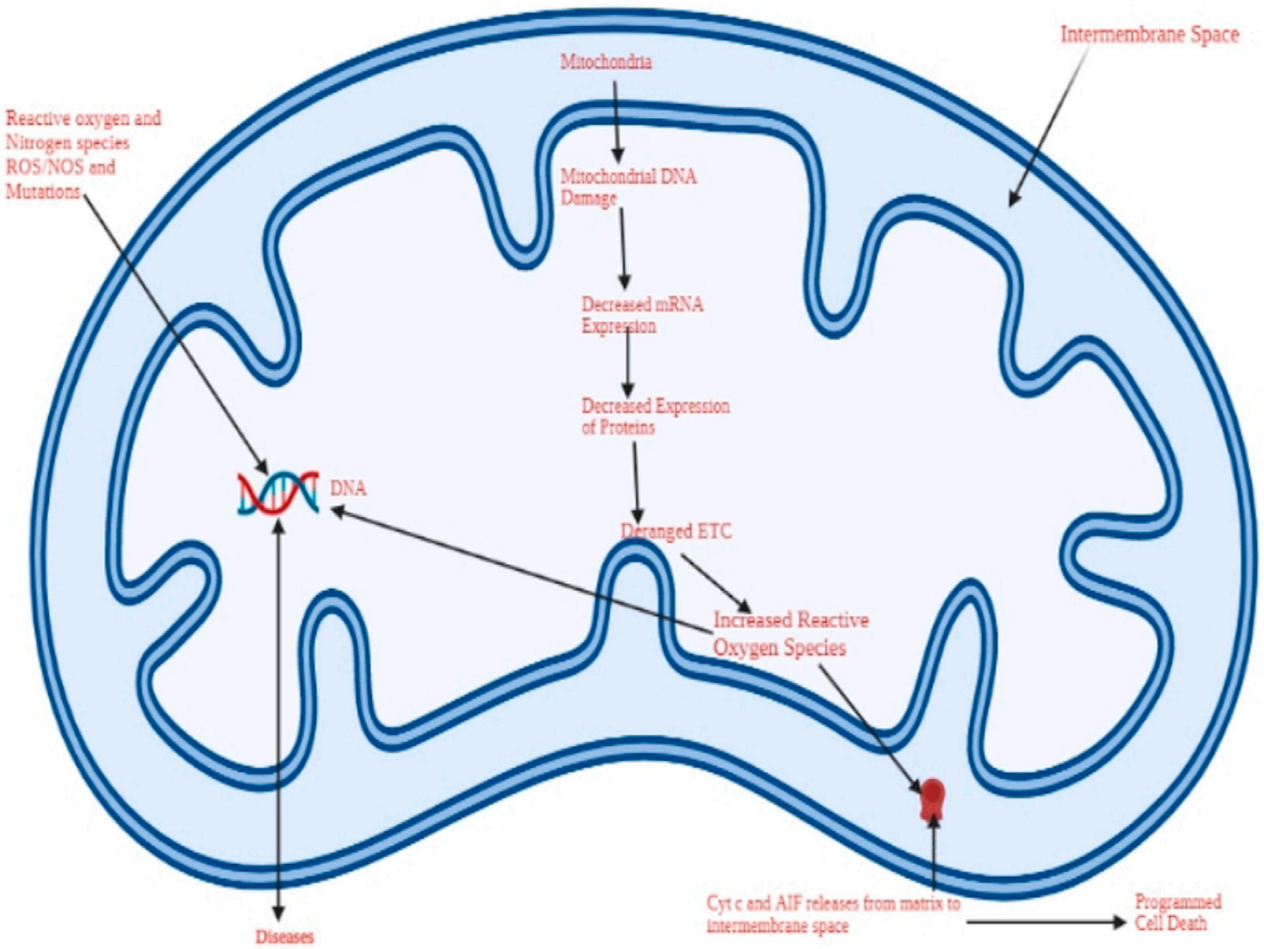
A schematic representation of oxidative stress and the resulting mitochondrial damage due to the reactive oxygen species. The cell dies through apoptosis, a process involving DNA damage, programmed cell death, and the activity of reactive oxygen species.

Oxidative stress refers to the imbalance between limited antioxidant defense mechanisms and instruments and the formation or rate at which the reactive oxygen species are formed (Conde de la Rosa et al., 2022). Figures 1, 2 illustrate oxidation resulting in cell death and disease occurrence. Figure 1 indicates that cell damage results from high production of ROS than antioxidants or antioxidant biomolecules. Figure 2 categorizes oxidation and cell damage by illustrating cell damage in mitochondrial cells. The association between Dexmedetomidine, oxidative stress and eventual cell death can be discussed based on the reported outcomes. Literature and previous studies indicated that Dexmedetomidine suppresses oxidative stress, a crucial process through which the three elements can be associated.

**FIGURE 3:**
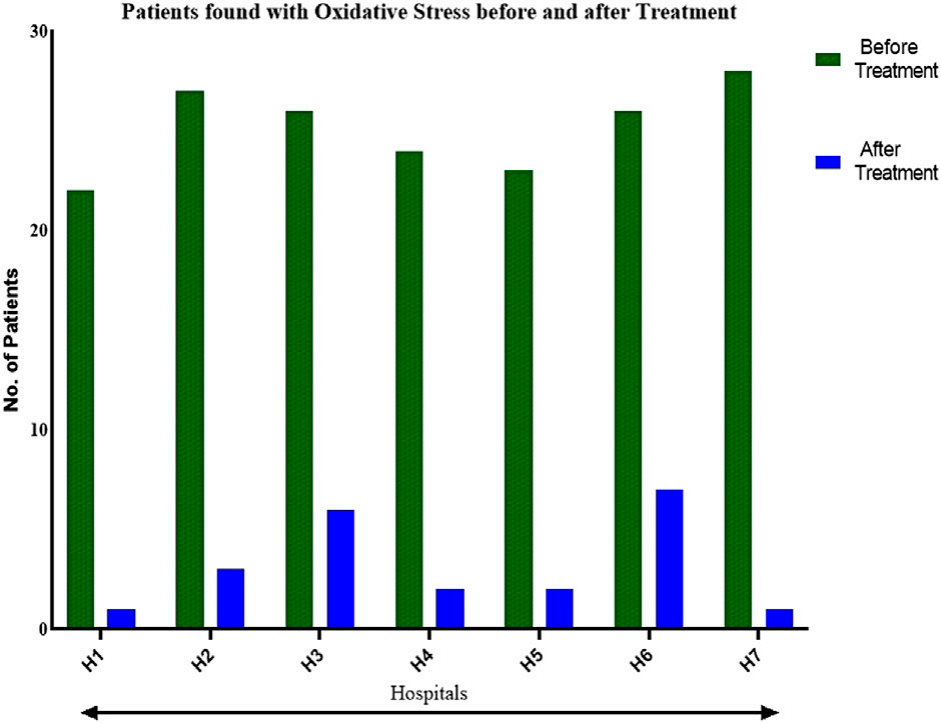
A comparison of the effects of Dexmedetomidine on oxidative stress before and after treatment, indicating the potency and clinical significance of the medication in primary liver cancer.

A direct association between Dexmedetomidine and oxidative stress and an indirect relationship between Dexmedetomidine and programmed cell death was established in Uchinda *et al.'s* study outcomes. Dexmedetomidine directly suppresses oxidative stress and indirectly inhibits programmed cell death through an anti-inflammatory mechanism. Dexmedetomidine’s suppressive functions inhibit inflammation, where inhibition of inflammation of hepatocytes downregulates apoptosis and the production of the reactive oxygen species. Indication of Dexmedetomidine implies less inflammation and, equally, the resulting inflammation. By this, the rate of oxidative stress and programmed cell death decreases. These are key observations made during hepatectomy since the nature of the cells removed by the surgical processes differs. Figure 3 shows the number of patients with oxidative stress before and after treatment. Dexmedetomidine intervention suppresses oxidative stress, suggesting Dexmedetomidine’s clinical significance. Based on these outcomes, Dexmedetomidine can reduce oxidation, decreasing cell death in primary liver cancer.

The significant reduction in oxidation reflects the potential clinical advantages of Dexmedetomidine among liver cancer patients. Supplementary Appendix Table SA1 includes the statistical data on reduced oxidation following dexmedetomidine intervention among cancer liver patients. These outcomes suggest the potential implementation of Dexmedetomidine against liver cancer for patients undergoing hepatectomy.

**FIGURE 4:**
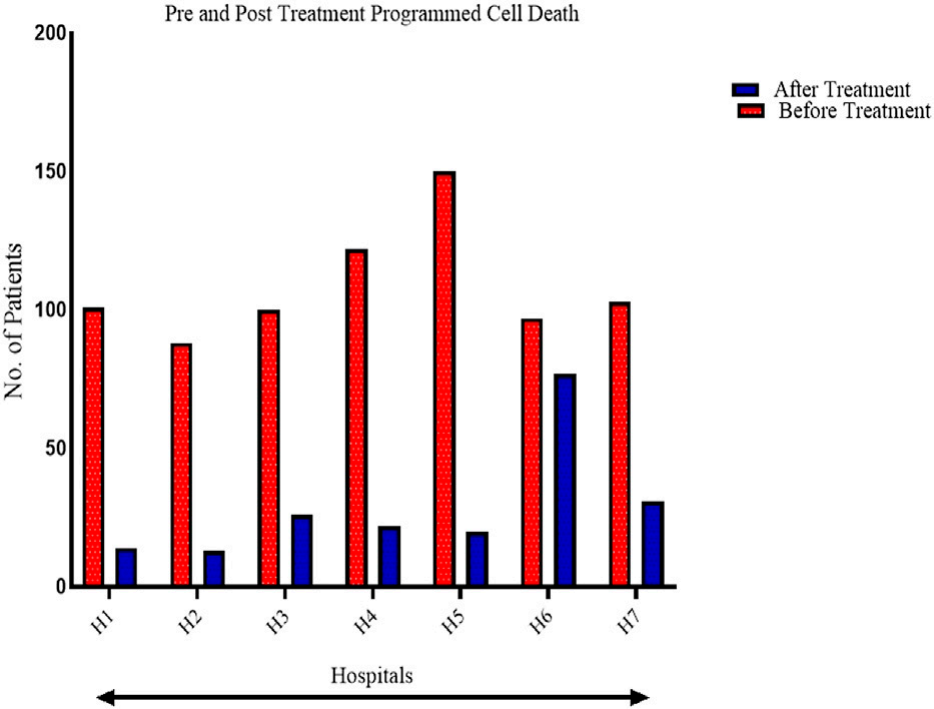
A comparison of pre-and post-treatment outcomes of Dexmedetomidine on programmed cell death: Apoptosis.

Assessment of patients with primary liver disease reveals the effect of Dexmedetomidine on oxidative stress and programmed cell death. After reviewing patients who had undergone hepatectomy following dexmedetomidine intervention and comparing the outcomes with the group that had not reviewed the treatment, we could tell the effect of Dexmedetomidine on oxidative stress and programmed cell death (Figure 4). Figure 4 shows a decline in the number of patients found with apoptosis following Dexmedetomidine, also suggesting Dexmedetomidine’s clinical importance; reduced cell death. The comparison shows that Dexmedetomidine decreased the intensity of cell death among patients with primary liver cancer. Additionally, we focused on evidence on the status of the cell, records, or reported outcomes concerning the reactive oxygen species, including the imbalance between the antioxidant processes and reactive oxygen compounds, alongside the reaction between the reactive oxygen species and the vital biological molecules and the DNA.

The statistical difference reported on post-treatment programmed cell death was quite significant. A consistent pattern in the decrease in the number of patients found with cell death was consistent in the seven hospitals. The statistical differences are captured in Supplementary Appendix Table SA2. The consistent decrease in cell death emphasizes Dexmedetomidine’s effects on overall outcomes among liver cancer patients undergoing hepatectomy.

### The effect of dexmedetomidine on liver function

Liver functions include the maintenance of blood thickness, filtering toxins, eradicating alcohol and other toxins, regulating blood sugar levels, and vitamin synthesis. Even before discussing the effect of Dexmedetomidine on liver functions, study outcomes on oxidative stress and programmed cell death shows that cell functions and impaired due to oxidative stress and cell death in the surgical group that did not receive dexmedetomidine intervention. We could tell that oxidative stress and programmed cell death impair liver functions through DNA damage and the interaction between the reactive oxygen species and macromolecules like lipids and proteins. In other words, the death of liver cells results in the loss of liver functions. It results in decreased antioxidant functions the liver performs, suggesting high oxidation.

Dexmedetomidine’s effect on liver functions is dependent on the structural element. By suppressing oxidative stress, restoration of liver functions is imminent as the structural elements are not damaged. The DNA, to begin with, facilitates liver functions by optimal command of hepatocytes. When damaged, coordination and control over hepatocytes are lost, leading to poor functions. Limiting H_2_O_2_-induced apoptosis is a key attribute of liver functions. Dexmedetomidine treatment inhibits H_2_O_2_-induced apoptosis, a crucial structural outcome of liver functions. Hepatocyte death through H_2_O_2_-induced apoptosis undermines functions. Establishing a balance between the produced reactive oxygen species and antioxidant functions supplements the anatomic feature.

Reactive oxygen species cause cellular dysbiosis—of which the interaction between the reactive oxygen species and important biomolecules in the cellular matrix-arises in the present study. Oxidation of proteins, lipids, and other important molecules in the cellular matrix undermines overall organ function. Dexmedetomidine administration downregulates the production of the reactive oxygen species, creating a balance between the reactive species and the antioxidant functions. Undoubtedly, cellular functions improve, implying an improvement in overall organ function.

**FIGURE 5:**
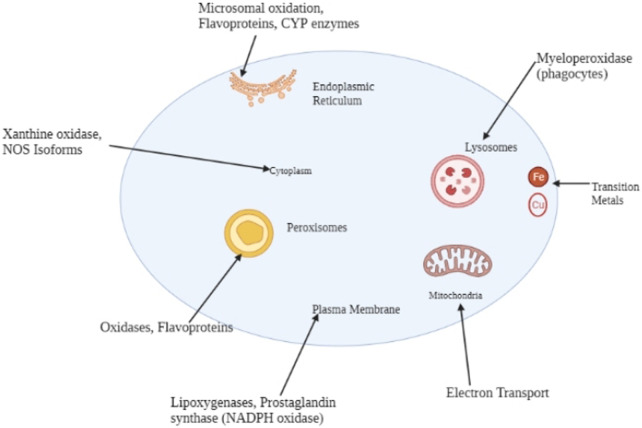
Major endogenous sources of reactive oxygen species include the endoplasmic reticulum, mitochondria, peroxisomes, the cytoplasm, and the plasma membrane.

Physiologically, liver functions are founded on homeostatic principles where the necessary anatomical structures perform their respective functions. Dexmedetomidine’s effects on liver functions depend on cellular functions and the status of the macromolecules performing different functions. Figure 5 shows the sources of the reactive oxygen species, the mitochondria.

Upon administration, Dexmedetomidine will suppress the oxidative processes in the mitochondrial matrix, implying an uninterrupted energy supply to hepatocytes. The energy supplied to hepatocytes is key to maintaining and up-stepping liver functions. Suppressed oxidative processes will spare macromolecules in the intracellular and extracellular matrix, enabling optimal cellular functions. The latter is a key viewpoint for cellular health and its consequential functions.

### Dexmedetomidine and expression of peripheral immune cells

**FIGURE 6:**
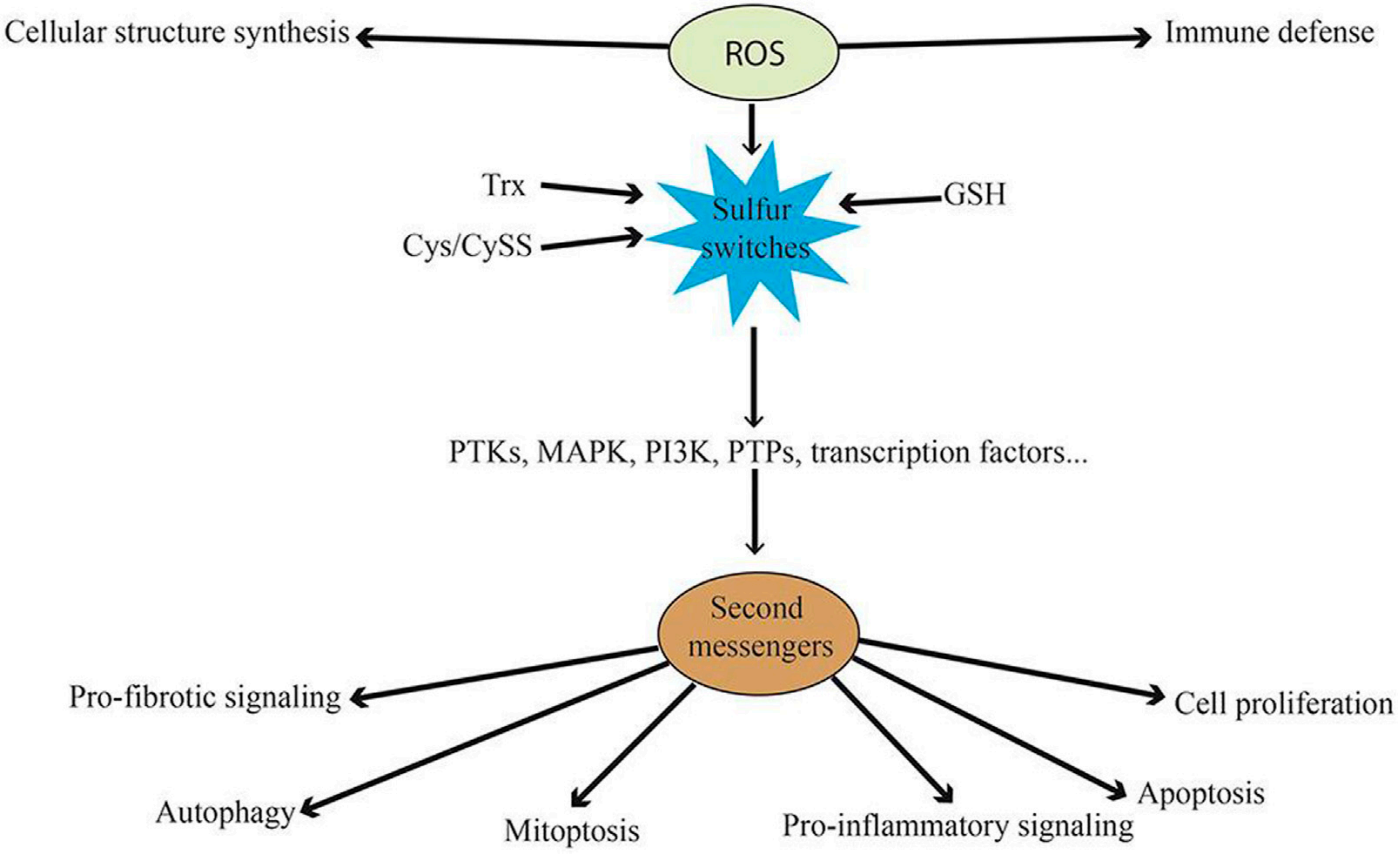
A schematic illustration of the mechanism through which the oxidative process facilitates disease progression. The reactive oxygen species interacts with immune cells and influences cellular structure synthesis, informing the basis of the disease progression pathway.

**FIGURE 7:**
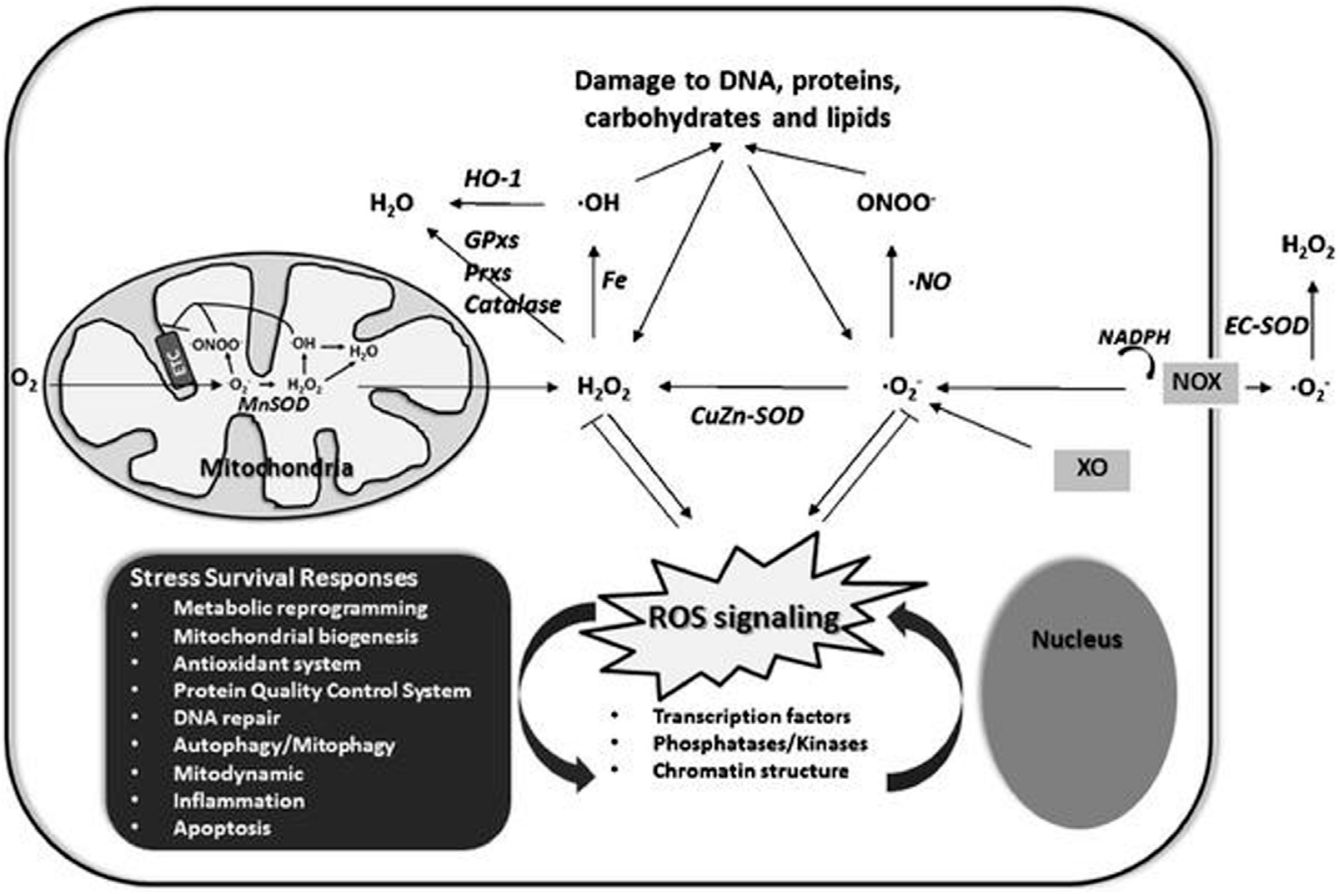
A schematic representation of redox homeostasis. Normal cell functions are at optimum levels when the ROS production and elimination rate are equal. An imbalance between the two, especially increased ROS production than elimination, results in oxidation and cell damage.

**FIGURE 8:**
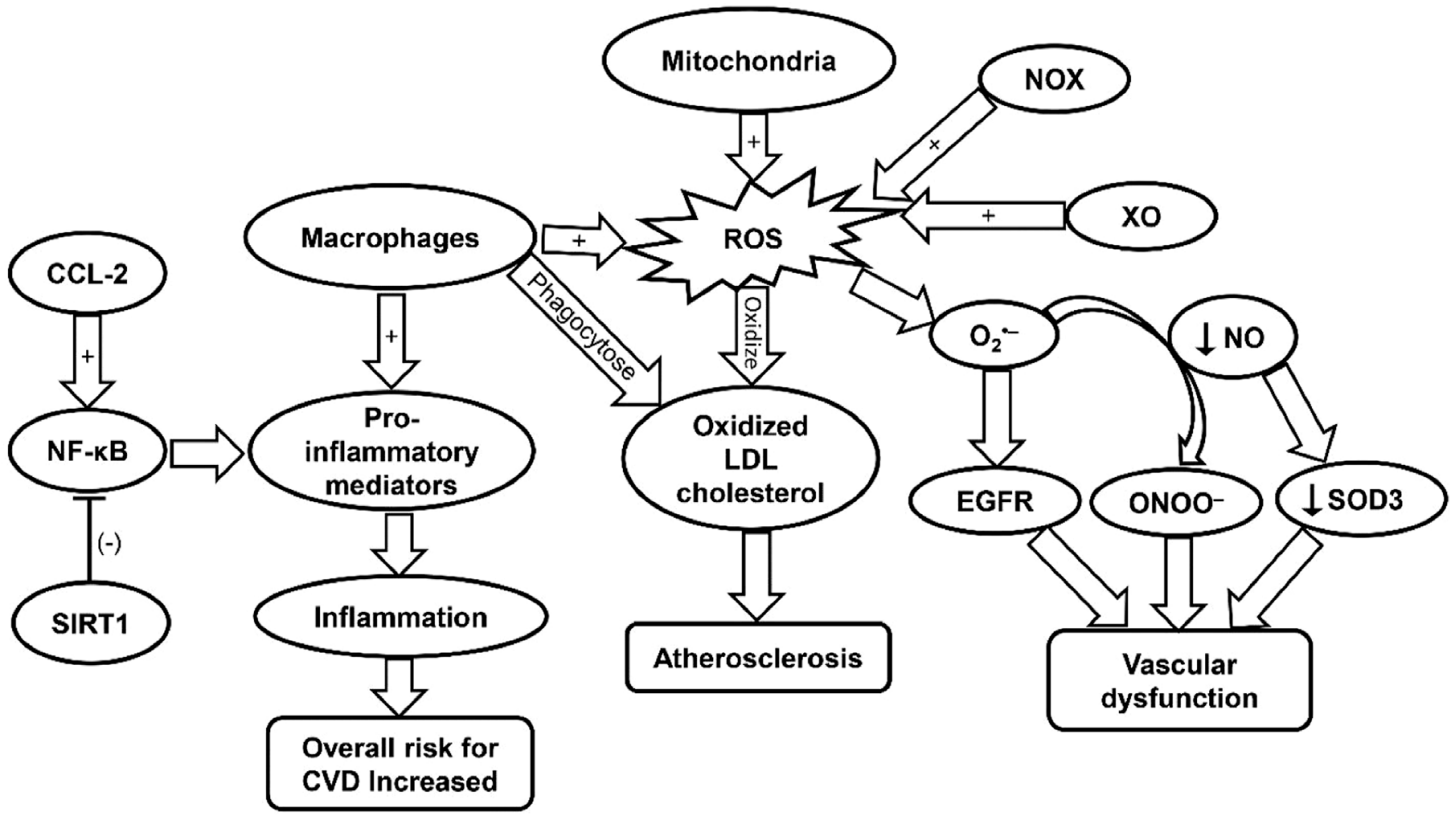
A schematic representation of the effect of distant tumor progressive factors, including Dexmedetomidine's effects on peripheral immune cells. Cytokines and chemokines play a significant role in the progression of primary liver cancer through various mechanisms.

The progression of primary liver cancer is a function of suppressed immune functions or cells. Primary liver cancer patients who had undergone hepatectomy had decreased expression of the peripheral immune cells. We reviewed blood test outcomes obtained from primary liver cancer patients who had undergone hepatectomy to establish the expression of the peripheral immune cells. Supplementary Appendix Table SA3 summarizes the expression of immune cells among patients, reported by the blood tests. The fundamental outcome of the review revealed the downregulation of immune cells among the patients (Figures 6, 7, 8).

## Discussion

### Dexmedetomidine’s effects on oxidative stress and programmed cell death

Oxidative stress, induced by the reactive oxygen species generated by the mitochondria, triggers inflammation, among other outcomes like hepatocyte damage, insulin resistance, and enhanced pathologic polyploidization (Fu and Chung, 2018). Also, oxidative stress is responsible for the migration and the subsequent invasion and metastasis of hepatocellular carcinoma. Uchinda *et al.* found that Dexmedetomidine indirectly limits apoptosis by suppressing oxidative stress. From this account, we could identify Dexmedetomidine’s fundamental mechanisms of action in primary liver cancer, suppressing oxidative stress, which prompts the connection with programmed cell death (García-Pras et al., 2021). The literature on oxidative stress indicates that an imbalance between the reactive oxygen species and the antioxidant biological processes yields inflammation, which subsequently causes cell death. Inflammatory cell death is the main effect of Dexmedetomidine, as reported by García-Pras *et al.* (García-Pras et al., 2021). The Supplementary shows the positive effects of Dexmedetomidine in liver cancer patients undergoing hepatectomy. The significant decrease in patients found with cell death and oxidation following dexmedetomidine treatment informs the clinical benefits and suggests the rationale for its use among liver patients undergoing hepatectomy.

Dexmedetomidine’s suppressive effects on oxidative stress initiate a cascade of biological processes that eventually inhibits programmed cell death through any of the previously mentioned processes (Figures 3, 4). In this case; apoptosis becomes the center of discussion as inhibited oxidative stress limits inflammation: Which is a key outcome and factor in programmed cell death and oxidative stress. Inflammatory cell death decreases following Dexmedetomidine’s suppression of oxidative stress. Consequently, the rate of cell death decreases. Dexmedetomidine’s effect on programmed cell death is indirectly achieved through oxidative stress. After comparing the pre-and post-treatment effects of Dexmedetomidine, we found a significant decline in the number of patients with oxidative stress and apoptosis (see Figures 3, 4). Dexmedetomidine’s suppressive effects on oxidative stress are reported to result in H_2_O_2_-induced apoptosis. Records of post-treatment hepatectomy on cell death show a decrease in the number of patients found with cell death, specifically apoptosis.

The discussion of the effects of Dexmedetomidine on oxidative stress and programmed cell death unfolds independently. As with oxidative stress, suppressed production of the reactive oxygen species downregulates the interaction with important biomolecules like proteins, deoxyribonucleic acid, and lipids. Conde de la Rosa et al. (2022) report that the interaction between the reactive oxygen species with these biomolecules results in cell death. Deoxyribonucleic acid and lipids are important biomolecules facilitating cellular functions. The interaction between these biomolecules and the reactive oxygen species impairs cellular functions.

At high levels, reactive oxygen species impair the physiological functions of cells through protein, lipid, and DNA damage. Additionally, the impairment of other macromolecules facilitating cellular functions results in health pathologies like liver cancer, alongside other complications, including neurodegenerative disorders and cardiovascular diseases (Rowe et al., 2008). Figure 5 shows the sources of the reactive oxygen species, including the same matrix where the compounds are likely to act. The reactive oxygen species are most likely to interact with the macromolecules, and the DNA within the mitochondrial matrix, thereby disrupting the energy supply to the cells. Additionally, Figure 2 illustrates mitochondrial damage due to the accumulation of reactive species within the cell. The cell dies through apoptosis.

We could deduce that Dexmedetomidine reduces programmed cell death by suppressing oxidative stress. Reducing programmed cell death follows a cascade of biological processes and chemical reactions involving or facilitating cellular functions. Figure 5 shows typical sources or biological spaces for generating reactive oxygen species. The DNA of the cells where the reactive oxygen species are produced is at risk of damage, implying cellular death. The suppressive effects of Dexmedetomidine indirectly lead to cellular death through oxidative stress.

Similarly, the probability of cellular death is courtesy of the interaction between reactive oxygen species and vital macromolecules like lipids and proteins. Figure 1 shows that reactive oxygen species trigger cell damage and eventually death. ROS production and the innate antioxidant processes inform disease status; high ROS production, with low antioxidant processes, leads to cell death and, eventually, disease. The converse of this phenomenon is true. Dexmedetomidine inhibits H_2_O_2_-induced apoptosis (Liu X et al., 2020), a representation of the effect of the medication in primary liver cancer, representing an induced antioxidant function. In this manner, Dexmedetomidine downregulates cell death through non-enzymatic mechanisms.

Clinical practice and investigations have reported the effect of Dexmedetomidine in primary liver cancer among patients undergoing hepatectomy. Weng et al. (2021) investigated the effect of Dexmedetomidine on oxidative stress and found that the drug inhibits H_2_O_2_-induced apoptosis: An observation reporting Dexmedetomidine’s dual effect. By inhibiting H_2_O_2_-induced apoptosis, Dexmedetomidine stands out as a suppressor of oxidative stress and programmed cell death (Cantu et al., 2011). The effect of Dexmedetomidine became clear after contrasting outcomes in the treatment group that underwent hepatectomy against the group that did not undergo the procedure. Surgical records show that patients who received dexmedetomidine treatment experienced less oxidative stress and cell death. Evidence of cell damage and the reaction between the important macromolecules, alongside DNA damage, was found to be less in the group treated with Dexmedetomidine than in the control group.

Participants in the treatment group did not report DNA damage. In contrast, participants who did not receive dexmedetomidine treatment had an imbalance in antioxidant processes and the produced reactive oxygen species, DNA damage, and high interaction between the reactive oxygen species and biological macromolecules like lipids and proteins. With reduced oxidative stress and cell death, there was minimal debris removed by the surgical procedure among primary liver cancer patients who received dexmedetomidine treatment. However, a true converse was observed in the patient group who did not receive dexmedetomidine treatment. We noticed that most surgical procedures were deliberated to remove dead cells from oxidative stress and programmed death. Low oxidative stress and programmed cell death are the foundation of contraindicating hepatectomy among patients with primary liver cancer.

### Effects of dexmedetomidine and liver function

Literature dexmedetomidine’s protective effects on the liver (Soleimanpour et al., 2019). The analysis of Dexmedetomidine’s effects on oxidative stress and programmed cell death holds substantial evidence supporting these claims. Dexmedetomidine suppresses oxidative stress by inhibiting H_2_O_2_-induced apoptosis, a crucial outcome concerning liver function. Another important effect of dexmedetomidine treatment concerns limiting programmed cell death resulting from the reactive oxygen species, where the production rate of reactive oxygen compounds surpassed the antioxidant mechanisms. The evidence and information from the discussion on programmed cell death and oxidative stress connect Dexmedetomidine to liver functions.

**FIGURE 9:**
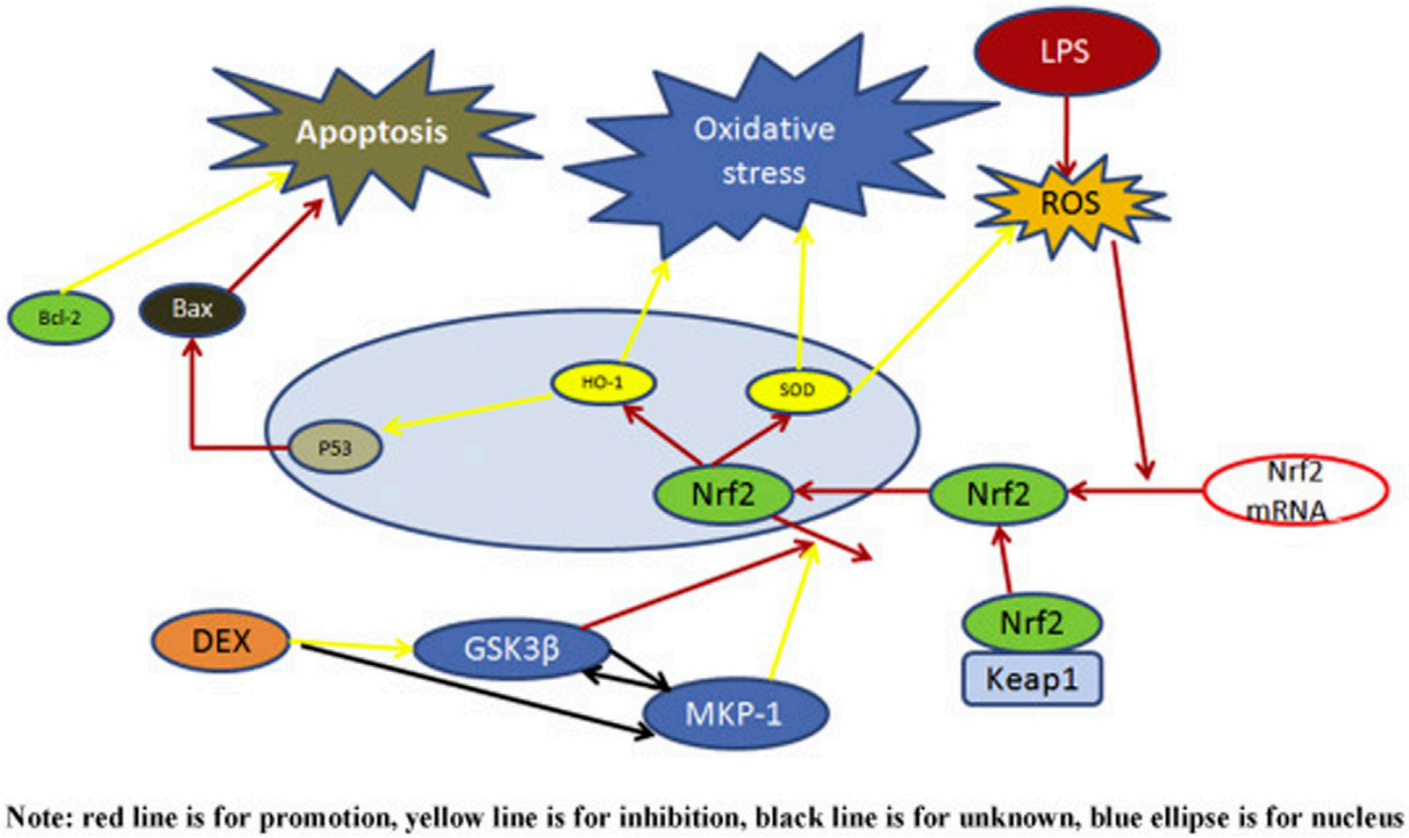
A schematic representation of Dexmedetomidine's protective effects on the liver. Dexmedetomidine attenuates lipopolysaccharides involved in the inhibition or downregulation of the activity of reactive species.

After finding that Dexmedetomidine suppresses oxidative stress, we concluded that the actions of the reactive oxygen species, like interaction with lipids and proteins, alongside DNA damage, significantly decreased. DNA damage, alongside the interaction between the reactive oxygen species and the vital macromolecules like lipids and proteins. Dexmedetomidine’s mechanism of action produces a protective effect on the liver (see Figure 9). Dexmedetomidine’s protective function results are based on autophagy, downregulation of p62, alongside upregulation of Beclin 1 and LC3II (Yu et al., 2021). The protective effects result from a series of biological processes and functions like suppression of oxidative stress. As mentioned earlier, Dexmedetomidine suppresses oxidative stress, reducing the programmed cell death rate. These processes reduce possible inflammation and hepatocyte damage, which result in poor liver function. These outcomes can be contrasted to Conde de la Rosa et al.'s account of how oxidation causes disease progression. Without intervention by Dexmedetomidine, the loss of liver functions is reversed, as reported by Yu et al. (2021).

Oxidative stress and programmed cell death threaten the cell and organ function. This observation agrees with the literature on reactive oxygen species, where structural abnormalities and malfunctions were reported (Cichoż-Lach and Michalak, 2014). Preservation of liver structures is key to optimum functions. Inhibition of oxidative stress is a key step in restoring liver functions, and reduced programmed cell death through limited H_2_O_2_-induced apoptosis, is a paramount step in improving liver performance. Arguably, liver functions are bound to improve when no hepatocytes die or are lost. Suppressing oxidative stress creates a conducive environment for the mitochondrial matrix to facilitate hepatocytes’ functions, amounting to improved liver function.

**FIGURE 10:**
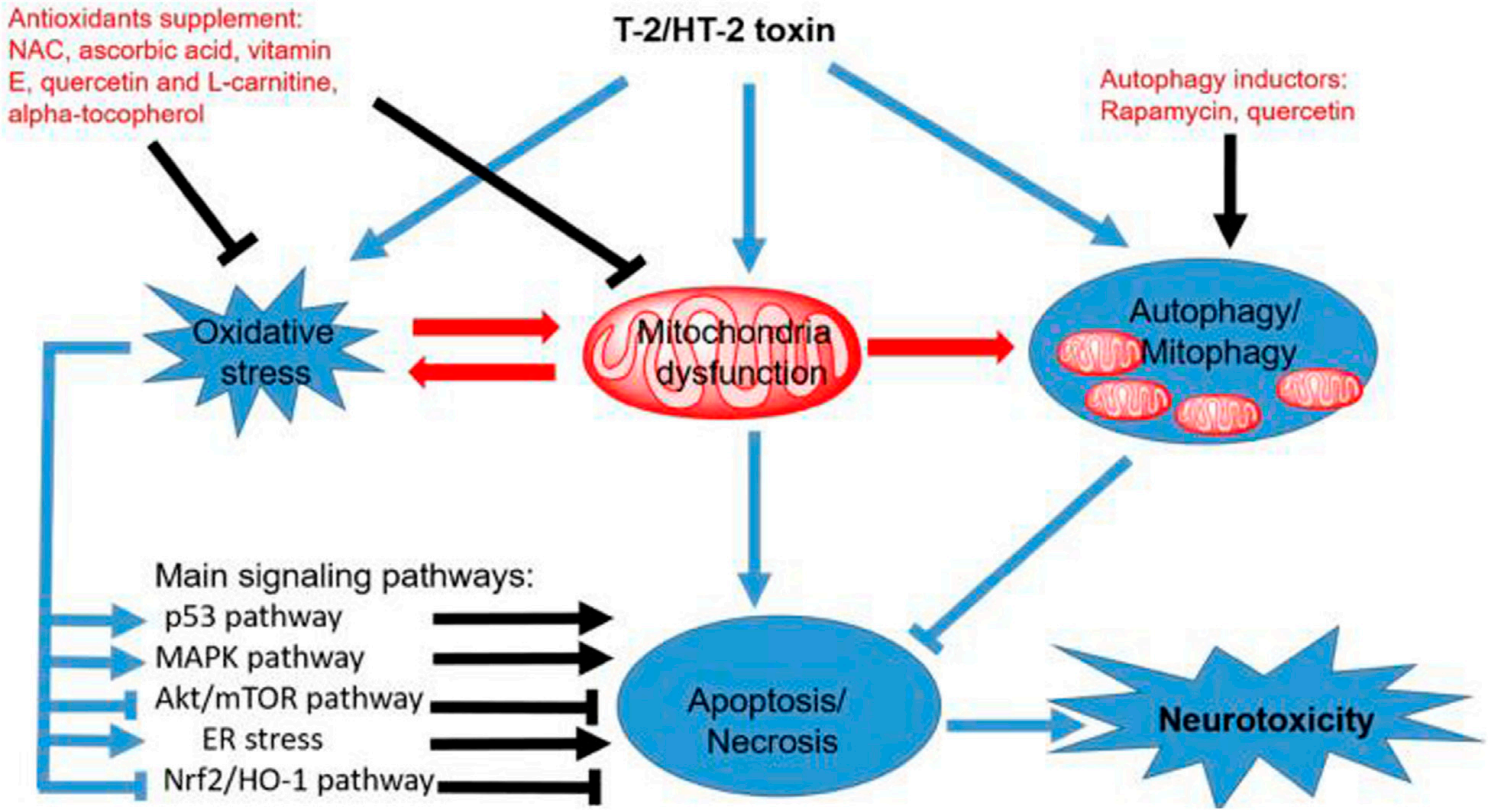
A schematic representation of oxidative stress. Mitochondrial dysfunction is central to the oxidative processes, implying that restoring mitochondrial functions and homeostatic requirements is crucial for optimum liver functions.

Figure 10 shows the oxidative stress mechanism against which Dexmedetomidine acts and restores liver functions. DNA damage, protein adduction, lipid peroxidation, infiltration of immune cells, activation of hepatic stellate cells, and inflammasomes, alongside mitochondrial dysfunction, are key processes impeding liver function (Li et al., 2015). All of these processes are functions of oxidative stress. We can confidently stress that reversing oxidative processes or suppressing oxidation of lipids and protein adduction are key steps in revamping liver functions. Oxidative stress impairs liver functions, and the converse is true: The effect of Dexmedetomidine improves liver functions (Li et al., 2015).

### Expression of peripheral immune cells

Supplementary Appendix Table SA3 summarizes Dexmedetomidine’s effects on immune cells. Chen et al. (2020) reported that Dexmedetomidine suppresses or induces immune cell functions, implying better expression. One study reported that Dexmedetomidine somewhat reduces the magnitude of immune function suppression, giving way to cancer development (Cai et al., 2022). The expression of peripheral immune cells is the downside of Dexmedetomidine among primary liver cancer patients undergoing hepatectomy.

Our findings agree with the literature on the effects of Dexmedetomidine concerning the expression of immune cells. B- and T-lymphocytes and monocytes are the main peripheral immune cells involved in primary liver cancer. We found poor expression of these cells among primary liver cancer patients who had received dexmedetomidine treatment than the control group, indicating a negative outcome. The literature on primary liver cancer and dexmedetomidine treatment shows tolerance to tumors, leading to the progression of the disease (Sachdeva et al., 2015; Sachdeva and Arora, 2020). The figure below shows that Dexmedetomidine induces proinflammatory cytokine (T-cells), a key process in oxidation in the development of primary liver cancer.

The induction of proinflammatory cytokine and chemokines (interleukin-2) is detrimental among primary liver cancer patients who have undergone hepatectomy (Sachdeva et al., 2015; Sachdeva and Arora, 2020; Cai et al., 2022; Chen et al., 2022; Lu et al., 2022). From the observations above, we could conclude that Dexmedetomidine could be a potential agent against the progression of primary liver cancer. The induction of proinflammatory cytokines and chemokine exacerbating liver cancer development changes the tune and indicates the detrimental effects of its intervention. Caution should be taken against dexmedetomidine administration as it could trigger the progression of primary liver cancer.

Characteristic immunological functions unfold following dexmedetomidine treatment. Cai et al. (2022) investigated the role of immune cells in the progression of primary liver cancer following dexmedetomidine treatment, where the tumor cells were found to escape immunosurveillance. This was a significant observation that could imply clinical practice recommendations for contraindication.

## Conclusion

Due to the health concerns raised by primary liver cancer, we analyzed the effect of a commonly-indicated medication, Dexmedetomidine, on primary liver cancer. Dexmedetomidine’s effects were studied concerning the primary biological processes involved in the progression of primary liver cancer: Oxidative stress, programmed cell death, expression of peripheral immune cells, and liver functions. The study was performed among primary liver cancer patients who had undergone hepatectomy, where results were compared with patients who had not undergone dexmedetomidine treatment.

In this article, we reviewed outcomes obtained from patients who had undergone hepatectomy to remove tumors and cells affected by cancer to tell the effect of Dexmedetomidine on oxidative stress and programmed cell death. In contrast to the control group, where Dexmedetomidine was not administered, the experimental group receiving dexmedetomidine treatment was found to have low oxidation and consequential programmed cell death (Figure 4). Dexmedetomidine suppressed oxidative processes in the cells. This outcome affected cell functions, death, and overall organ outcomes. Post-treatment outcomes show a decrease in patients with oxidative stress (Figure 3).

The antioxidant effects of Dexmedetomidine countered the imbalance between the biological antioxidant processes and the production of reactive oxygen species. Normally, oxidative stress results from an imbalance between the production of reactive oxygen species and the biological antioxidant processes, where the latter is exceeded by the latter. Accumulation of highly reactive species accumulates in the intracellular and extracellular spaces, damaging the DNA of the cells within these spaces (Figure 10). Additionally, the reactive species interact with the macromolecules facilitating biological processes like lipids and proteins, all of which result in cell death.

Dexmedetomidine administration suppresses oxidation, reversing the DNA damage and the interaction between the reactive oxygen species with lipids and proteins. Homeostasis is restored in the intracellular and extracellular spaces, which improves organ function. The literature and our study findings show that dexmedetomidine administration improves liver functions by suppressing oxidation. Dexmedetomidine’s antioxidant effects prevent cell death associated with oxidation. Additionally, Dexmedetomidine was directly associated with reducing hydrogen peroxide-induced apoptosis. These outcomes are key in managing primary liver cancer as they yield positive outcomes. In contrast to the control group, participants in the intervention group had fewer tumor cells. Improved liver functions were a common finding among the patients who received Dexmedetomidine.

We found adverse effects of Dexmedetomidine among primary liver cancer undergoing hepatectomy. After reviewing the expression of immune cells, we noted less or reduced expression of tumor-suppressing immune cells. Instead, proinflammatory cytokines and chemokines were extensively expressed. The latter is associated with the development of cancer. These outcomes indicate the detrimental effects of dexmedetomidine intervention among primary liver patients. A review of the surgical records indicates that patients in the intervention group developed tumors and were found with tumor progression, courtesy of the increased expression of proinflammatory cytokines and chemokines. This observation extends oxidative stress, associated with high inflammatory outcomes in primary liver cancer.

Based on these outcomes, clinical practices should involve caution to avoid adverse effects. Primary liver patients undergoing hepatectomy should avoid Dexmedetomidine for safety and efficacy purposes as the tumor progression is likely to arise from the expression of proinflammatory cytokine and chemokines. Arguably, Dexmedetomidine’s effects unfold sequentially: Reduction of oxidation, reduction of cell apoptosis, downregulation of the expression of peripheral immune cells, and improved liver functions.

Liver functions are enhanced through hepatocyte regeneration following dexmedetomidine administration. Different immune cells are engaged in the three stages of hepatocyte regeneration, leading to enhanced liver function. Arguably, hepatocyte regeneration might not occur without dexmedetomidine intervention. Future studies should investigate Dexmedetomidine’s effects on the expression of immune cells to trigger the generation of liver cells.

## Data Availability

The original contributions presented in the study are included in the article/Supplementary Material, further inquiries can be directed to the corresponding author.
