# Supplementary material for: Effects of dexmedetomidine on oxidative stress, programmed cell death, liver function, and expression of peripheral immune cells in patients with primary liver cancer undergoing hepatectomy
Source: Front Physiol. 2023 Apr 11;14:1159746. doi: 10.3389/fphys.2023.1159746 (PMC10126774; doi:10.3389/fphys.2023.1159746)
Supplement: Supplementary file 1 [file Table1.docx]

| **Hospital** | **Patients (*n*) found with oxidation before treatment with Dexmedetomidine** | **Patients (*n*) found with oxidation after treatment with Dexmedetomidine** |
| --- | --- | --- |
| H1 | 22 | 1 |
| H2 | 27 | 3 |
| H3 | 26 | 6 |
| H4 | 24 | 2 |
| H5 | 23 | 2 |
| H6 | 26 | 7 |
| H7 | 28 | 1 |
