# Supplementary material for: Effects of dexmedetomidine on oxidative stress, programmed cell death, liver function, and expression of peripheral immune cells in patients with primary liver cancer undergoing hepatectomy
Source: Front Physiol. 2023 Apr 11;14:1159746. doi: 10.3389/fphys.2023.1159746 (PMC10126774; doi:10.3389/fphys.2023.1159746)
Supplement: Supplementary file 2 [file Table2.docx]

| **Hospital** | **Patients (*n*) found with cell death before treatment with Dexmedetomidine** | **Patients (*n*) found with cell death after treatment with Dexmedetomidine** |
| --- | --- | --- |
| H1 | 101 | 14 |
| H2 | 88 | 13 |
| H3 | 100 | 26 |
| H4 | 122 | 22 |
| H5 | 150 | 20 |
| H6 | 97 | 77 |
| H7 | 103 | 31 |
