# Supplementary figures and images for: Effects of dexmedetomidine on oxidative stress, programmed cell death, liver function, and expression of peripheral immune cells in patients with primary liver cancer undergoing hepatectomy
Source: Front Physiol. 2023 Apr 11;14:1159746. doi: 10.3389/fphys.2023.1159746 (PMC10126774; doi:10.3389/fphys.2023.1159746)

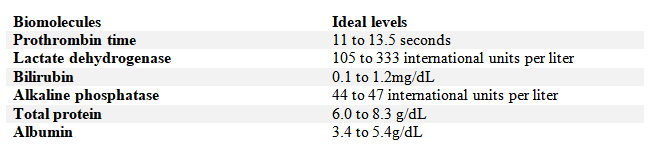

Supplement: Supplementary file 3 [file Table3.docx]
